# The species coalescent indicates possible bat and pangolin origins of the COVID-19 pandemic

**DOI:** 10.1038/s41598-023-32622-4

**Published:** 2023-04-05

**Authors:** Jialin Yang, Michael Skaro, Jiani Chen, Duna Zhan, Leke Lyu, Skylar Gay, Ahmed Kandeil, Mohamed A. Ali, Ghazi Kayali, Kateryna Stoianova, Pensheng Ji, Magdy Alabady, Justin Bahl, Liang Liu, Jonathan Arnold

**Affiliations:** 1 Statistics Department, University of Georgia, Athens, GA USA; 2 Institute of Bioinformatics, University of Georgia, Athens, GA USA; 3 Center for the Ecology of Infectious Diseases, University of Georgia, Athens, GA USA; 4 Genetics Department, University of Georgia, Athens, GA USA; 5 National Research Centre, Cairo, Egypt; 6 St. Jude Children’s Research Hospital, Memphis, TN USA; 7 Human-Link DMCC, Dubai, UAE; 8 University of Texas School of Public Health, Houston, TX USA; 9 Georgia Genomics and Bioinformatics Center, University of Georgia, Athens, GA USA; 10 Plant Biology Department, University of Georgia, Athens, GA USA; 11 Department of Infectious Diseases, College of Veterinary Medicine, University of Georgia, Athens, GA USA; 12 Department of Epidemiology and Biostatistics, College of Public Health, University of Georgia, Athens, GA USA

**Keywords:** Computational biology and bioinformatics, Evolution, Diseases

## Abstract

A consensus species tree is reconstructed from 11 gene trees for human, bat, and pangolin beta coronaviruses from samples taken early in the pandemic (prior to April 1, 2020). Using coalescent theory, the shallow (short branches relative to the hosts) consensus species tree provides evidence of recent gene flow events between bat and pangolin beta coronaviruses predating the zoonotic transfer to humans. The consensus species tree was also used to reconstruct the ancestral sequence of human SARS-CoV-2, which was 2 nucleotides different from the Wuhan sequence. The time to most recent common ancestor was estimated to be Dec 8, 2019 with a bat origin. Some human, bat, and pangolin coronavirus lineages found in China are phylogenetically distinct, a rare example of a class II phylogeography pattern (Avise et al. in Ann Rev Eco Syst 18:489–422, 1987). The consensus species tree is a product of evolutionary factors, providing evidence of repeated zoonotic transfers between bat and pangolin as a reservoir for future zoonotic transfers to humans.

## Introduction

Despite intense interest and focused investigations, the origins and evolution of the Severe Acute Respiratory Syndrome Coronavirus 2 (SARS-CoV-2) remain unresolved^1–4^. Linking of pathogen phylogenetic history with microevolutionary processes, such as drift, recombination, gene flow, and selection across gene and species trees may reveal insights into conditions for disease emergence^5^. This approach was used to trace the origin of HIV global epidemic, 2009 H1N1 influenza virus pandemic and SARS coronavirus through existing genome sequences in phylogenetic analysis^6–8^. Comparative genomic analysis of viral samples provide a historical view of the viral spread in much the same way that names in a historical record allow us to track our own ancestors^9,10^.

Understanding the origins of the SARS-CoV-2 pandemic can reveal insights into the mechanisms of interspecies transmission and disease emergence in general. This is important for pandemic risk mitigation. However, the complex history of viral interspecies transmission and recombination revealed by the conflicting phylogenetic signals can confound inferential methods^8,11–13^. Analysis of SARS-CoV-2 genomes have led to multiple phylogenetic hypotheses involving emergence from a reservoir species through intermediate hosts, such as the pangolin^14^ or other wild animals (bats, or another unsampled)^2,3^. In this study, using all available SARS-CoV-2 sequence data (up to April 1, 2020), other closely related beta coronaviruses and newly sequenced Middle Eastern Respiratory Syndrome (MERS-CoV) viruses, we investigate the deep phylogenetic relationships across gene trees^4,15^. Each gene tree reflects different histories of selection, gene flow, recombination and lineage sorting as the genes move across species boundaries, and as a consequence the gene trees between closely related species may be discordant across species boundaries^5^. To resolve these discordances, a consensus species tree was reconstructed from the 11 gene trees with their unique evolutionary histories. The species tree will permit inference about the origin of SARS-CoV-2 from animal reservoirs and provide a framework for classification of human SARS-CoV-2 mutations as they arise and spread.

## Results

### Different gene trees in SARS-CoV-2 have different histories across species boundaries

To develop a classification system for the Covid-19 samples, one approach is to use one gene tree, such as for the gene (S) encoding the Spike protein^16^. The limitation of this approach, when applied to a recently arisen species or subspecies, is that the resulting gene trees may percolate through the species tree in different ways and be discordant^5,17^. The classification will vary with the gene chosen due to a gene’s distinct evolutionary history under random drift, recombination, gene flow, and selection. The discordance of gene genealogies is then predicted from coalescent theory by virtue of genes having different paths through the organismal pedigree and provides a framework for understanding and analyzing these discordancies in the context of a species tree derived from individual gene trees^18^.

**Figure 1:**
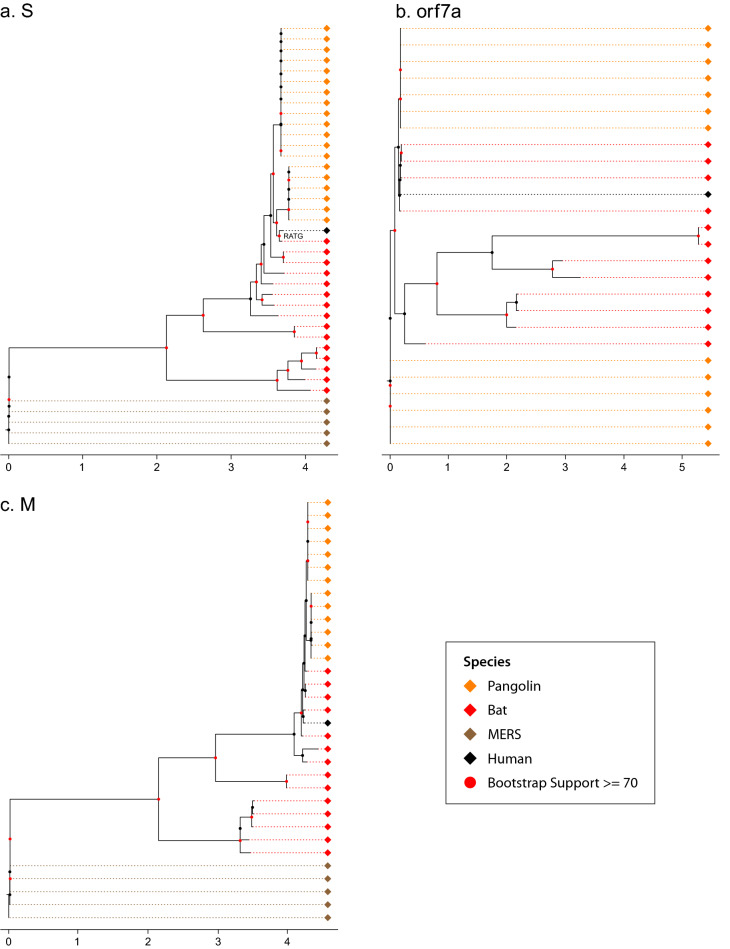
The consensus gene trees of the S, ORF 7a, and M genes are discordant: (**A**) S; (**B**) ORF 7a; (**C**) M. The gene trees were reconstructed by the method of maximum likelihood^19^ from 5,248 viral genomes. The consensus gene trees were reconstructed from a bootstrap sample of 100 gene trees. Human, bat, pangolin, and MERS-CoV coronavirus sequences are color-coded as black, pink, orange, and dark orange, respectively. The MERS CoV genomes root the gene trees. All 11 gene trees for the coronavirus genome are in the supplement (Figs. S1–S11). The black diamond represents the amalgamation of human SARS-CoV-2 lineages. A common scale bar for tree branch lengths is provided. The branch lengths on Fig. 1 are the mean of the expected number of substitutions per site across all 100 gene trees in the bootstrap sample of each gene (see Materials and Methods). Red dots on the trees indicate nodes with at least 70% bootstrap support.

Gene trees for up to 11 genes shared by the human, bat, and pangolin coronaviruses were reconstructed by the method of maximum likelihood^19^ (Figs. S1–S11 in the Supplement) (see Materials and Methods). Three of these gene trees are shown (Fig. 1). For the gene encoding the Spike (S) glycoprotein, there is a bat coronavirus lineage (RaTG13) close to the human coronavirus clade. The human clade is monophyletic with 100% bootstrap support, and the pangolin clade is paraphyletic (Fig. 1a). An examination of the ORF 7a gene tells a quite different story (Fig. 1b).

While the human clade remains monophyletic, the bat and pangolin clades are polyphyletic. For example, some bat coronavirus lineages are closer in descent to some pangolin coronavirus lineages than to other bat coronavirus lineages, while some pangolin coronavirus lineages are closer to some bat coronavirus lineages than other pangolin coronavirus lineages (polyphyly). This polyphyly of bat coronaviruses not only includes the RaTG13 bat lineage in China, but also for Cambodian bat coronavirus lineages. An examination of the gene (M) encoding the Matrix glycoprotein tells a third story (Fig. 1c). Here human and bat coronavirus lineages form a monophyletic group. The discordant evolutionary histories confound efforts to understand the origins of the SARS-CoV-2 pandemic. The conflicting phylogenetic signal in the gene sequences suggest that an alternative reconstruction method should be explored.

### Reconstruction of the species tree for human, bat, pangolin and MERS coronaviruses

**Figure 2:**
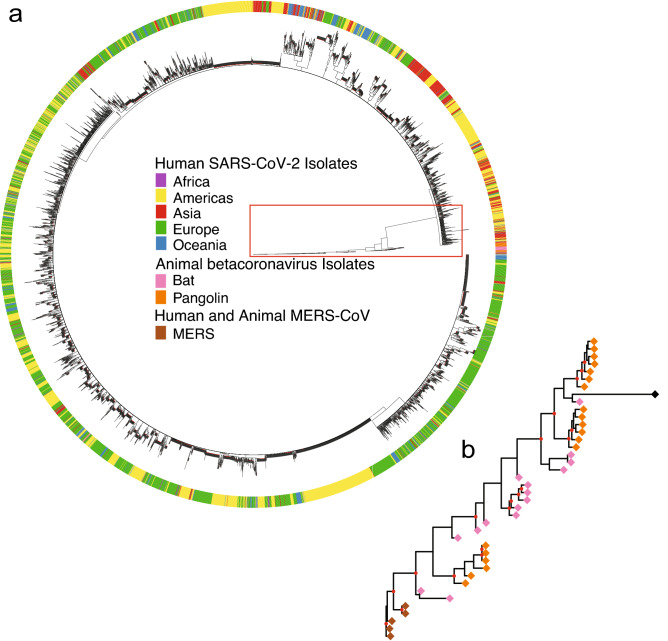
Consensus species tree of human, bat, and pangolin coronaviruses resolves the different histories of 11 gene trees in the coronavirus genome and was constructed by two methods (see Materials and Methods) from 5,248 viral genomes. (**a**) Using a distance based neighbor-joining method (Materials and Methods), the 5000 + taxa are represented in a circular consensus species tree. The outer circle designates regions. Each of the regions (Africa, Americas, Asia, Europe, and Oceania) in the consensus species tree have 80% of their viral genomes identified from that region or 80% bootstrap support and at least 50 viral genomes. (**b**) A blowup of the pangolin and bat coronavirus clades. Red dots indicate bootstrap support of at least 70%.

The species tree was reconstructed with a method developed here from the 11 individual gene trees to resolve the observed conflicting gene genealogies (see Materials and Methods). Not only does the species tree in Fig. 2a resolve the incongruent histories of gene trees, but the species tree is very informative about the history of natural variation across species boundaries. Notably, the human coronavirus clade is monophyletic with the amount of sequence variation between human coronavirus lineages less than 1/3 of that between bat and pangolin coronavirus lineages. The bootstrap support for the human coronavirus clade is 100%.

In the circular species tree we can see the variation of the bat, pangolin, and MERS coronavirus clades relative to the human coronavirus clade (Fig. 2a). One of the bat coronavirus lineages stands out quite close to the human coronavirus clade (blowup of pangolin and bat clades in Fig. 2b)^2^. The bootstrap support of the bat coronavirus lineage (RaTG13) from Yunnan province with the human coronavirus lineages is about 42%. This suggests a bat origin of the human coronavirus, which is further supported by more recent sampling of Cambodian bat coronaviruses^20^. A more detailed version of Fig. 2b with the bootstrap support values can be found in supplement Fig. S12, and the robustness of this finding with the addition of viral genomes is discussed below. Additional viral isolates from bats from Asia would be informative on the bat origin.

Another striking feature in Fig. 2a is the geographical structure in the tree. The Americas, Asia, and Europe are highly coherent in their clustering across the human coronavirus tree. There are at least 9 regional clades in the human coronavirus observed. This pattern emerged early in the pandemic, suggesting that founder populations quickly established and spread in each region. Continued global migration of lineages may result in competitive dynamics that could influence variant emergence.

The pangolin and coronavirus lineages are polyphyletic in the species tree. There are two clades within the pangolin coronaviruses with 77% support. These two clades contain distinct bat coronavirus lineages. One of these pangolin coronavirus clades contains the bat coronavirus (RaTG13) and human coronavirus lineages. The other pangolin coronavirus clade contains pangolin and remaining bat coronavirus lineages. The fact that the pangolin and bat coronavirus clades are still polyphyletic is consistent with the degree of variation relative to the human coronavirus lineages—a factor of at least 3X more than among human lineages.

As a further validation of this tree, the consensus species tree was regenerated with a second download of early coronavirus sequences totaling 40,028 human genome sequences and 50 other genome sequences in animal hosts, using a rebuild of the 11 gene trees by the method of maximum likelihood. The outcome was the same as in Fig. 2b, if the ORF 10 sequences were set aside. The rationale for excluding ORF 10 sequences is their absence from all bat and MERS sequenced genomes.

### Origin of human coronaviruses

With the construction of the consensus tree for human, bat, and pangolin coronaviruses, it is possible to reconstruct the ancestral sequence of the human SARS- CoV-2 coronavirus. The reconstructed ancestral sequence only differs by two nucleotides from that of the Wuhan sample (WH01), and these two nucleotide differences exist in ORF 1a-b at positions 6703C (A in WH01) and 11499T (A in WH01). This is consistent with a recent introduction to humans likely occurring in Wuhan.

The consensus tree (Fig. 2a) also predicts that the RaTG13 bat coronavirus lineage is closest to all human coronaviruses, suggesting a zoonotic transfer from bat, but the polyphyletic relation of the bat RaTG13 sequence with coronavirus isolated from pangolin suggests additional interspecies transmissions may have occurred in evolutionary history, leading to the emergence of SARS-CoV-2 in humans from bat. Microbial genomes, such as SARS-CoV-2, may require a new view of evolution involving pangenomes, in which viral genomes are connected in a network of genomes undergoing horizontal gene transfer events and other evolutionary processes^21^. If the bat origin held up under this alternative view to modification by descent in Fig. 2, then this would be a stronger validation of the bat origin hypothesis. To cross-validate this finding, a super network reconstruction from the 11 individual gene trees (see Materials and Methods) was performed with the same result—the RaTG13 bat coronavirus came out closest to that of human coronaviruses (Fig. S13). The network is without a root, but the lack of reporting of human SARS-CoV-2 prior to the Pandemic provides the additional piece of data to infer directionality of viral transfer.

The RaTG13 bat coronavirus sample was one of the closest to three human coronaviruses in China, Europe, and US and separated by 8–9 nodes from each of these sample in the super network (Fig. S14). Southeast Asia is a hotspot for biological diversity in 49 Rhinophilus bat species, hosting a variety of coronaviruses including those with a high similarity to SARS-CoV-2. Additional isolates in Southeast Asia provides additional sequence support for zoonotic transfer from bat or an intermediary, such as the pangolin^22^. Another Yunnan province bat coronavirus sample (RmYN02) is separated by 11–13 nodes from the nearest human coronavirus sequence (Fig. S13). Timing of the introduction into the human host was performed with a sensitivity analysis (Fig. S23) under varied hypotheses about the molecular clock. Support for varied hypotheses about the molecular clock from the BEAST analysis are examined (Table S2).

One further test of the bat origin was carried out with an alternate method of network reconstruction (RF-Net 2) designed for a reticulate view of evolution^23^. The number of reticulations was preset to 2 or 10. The RaTG13 bat coronavirus sample remained adjacent to the human coronavirus clade (Fig. S24), and the reticulation only occurred between bat and pangolin coronaviruses.

### Process affecting SARS-CoV-2 lineages as revealed by the coalescent

There are several interesting features about the variation in nucleotide divergence across species. The first is that the variation within bat coronaviruses dwarfs the variation within human, pangolin, and MERS coronaviruses. This is consistent with the whole bat coronavirus clade containing the human and pangolin coronavirus lineages (Figs. 2 and S15). These differences within and between species provide timing information about the processes generating the consensus species tree from the molecular clock.

For the purpose of inferring process from the consensus species tree, Bayesian species tree, inference was conducted on human coronavirus (10 strains randomly selected from ~ 40,000 human coronavirus), bat coronavirus (26 strains), pangolin coronavirus (19 strains), and MERS coronavirus (5 strains) using StarBeast^24^.

**Figure 3:**
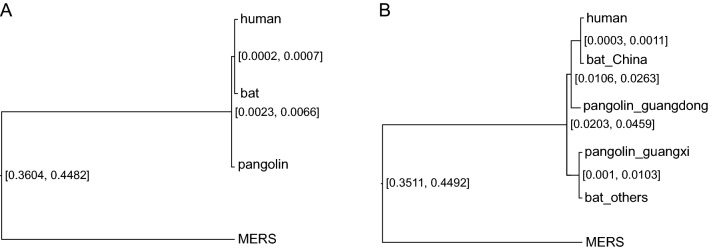
Bat and pangolin coronaviruses from the same location are more closely related than those from elsewhere. (**a**) The Bayesian species tree for human, bat, pangolin, and MERS coronavirus from StarBeast (Materials and Methods). (**b**) The Bayesian species tree for human, bat_China, bat_others, pangolin_guangdong, pangolin_guangxi, and MERS coronavirus from StarBeast. The numbers at the internal nodes of the species tree are 95% highest posterior density (HPD) interval for the depth of the internal node. The lower bound on the time of divergence of bat, human, and pangolin coronaviruses is (.0023/3)/(0.0002) = 4 year, using a 1/3 of a year as the length of the early pandemic as a calibration point for the lower bound in variation of 0.0002 in humans in panel A. The time of divergence of bat, human, and pangolin coronaviruses is (0.0041/3)/(0.0004) = 3.42 year, using a 1/3 of a year as the length of the early pandemic and the posterior mean branch lengths of the (pangolin, (human, bat)) node of .0041 and the (human, bat) node of 0.0004.

The 95% highest posterior density (HPD) interval [0.0023, 0.0066] for the height of the ancestral node, ((human, bat), pangolin), (see Fig. 3a) and height of the ancestral node, (human, bat), of [0.0002, 0.0007] indicate that the divergence time of bat coronavirus and pangolin coronavirus is 2.52 = (0.0034/0.00045)x (1/3) year under the assumption that a calibration time for the Beta coronavirus of 1/3 year for the length of the pandemic. Compared with the divergence time ~ 70 M year^25^ of bat and pangolin, the bat coronavirus and pangolin coronavirus were diverged more recently, indicating that there was gene flow (*i.e*., transmission between animals) between bat coronavirus and pangolin coronavirus after the divergence of species bat and pangolin. These results are similar with the BEAST analysis (see Materials and Methods) of a human coronavirus common ancestor of 60 y (Figs. S16–S21) and dating from the collection time of human samples (Fig. S22) with the caveat these single gene tree analyses appear to overestimate the divergence time between species.

To test directly for gene flow between bat and pangolin coronaviruses, these coronaviruses were divided into two groups by locality, those within China and those elsewhere for the bats and those within Guangdong province and those within Guangxi province for pangolin (Fig. 3B). The result was that (bat_China, pangolin-guangdong) and (bat-others, pangolin-guangxi) form two monophyletic groups in the species tree generated from the StarBeast analysis for the location-based sequence data. Bat and pangolin coronaviruses from the same locality were more closely related than bat and pangolin coronaviruses from different localities. This result also indicates that there is gene flow *(i.e*., transmission between animals) between bat coronavirus and pangolin coronavirus. The posterior probabilities of the 2 monophyletic clades in the StarBeast analysis are reported in Fig. S25.

### What coronavirus mutations occur along the S gene and what mutations are shared between human, bat, and pangolin coronaviruses in the S gene?

**Figure 4:**
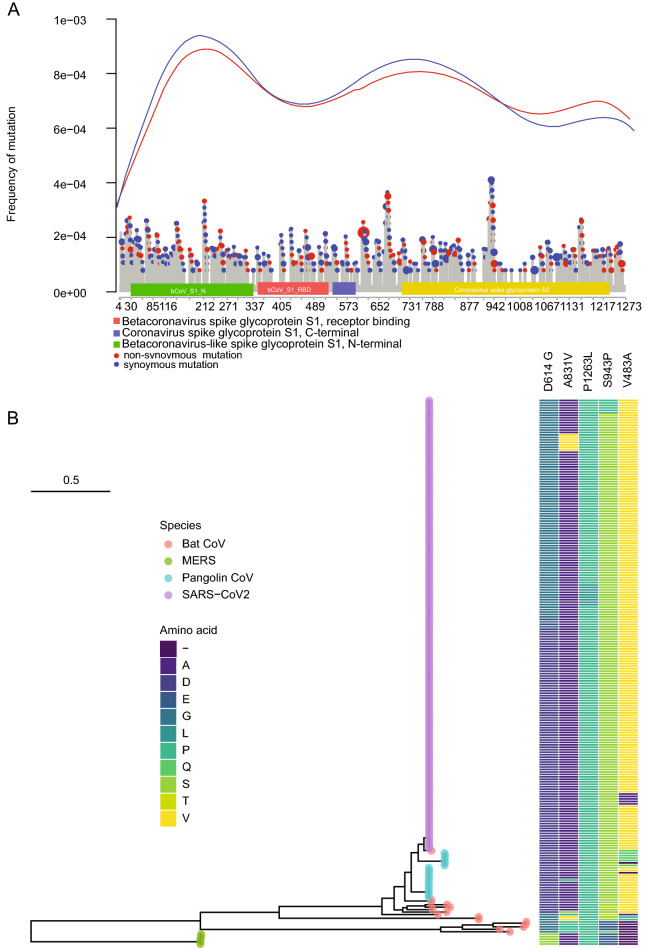
Particular nonsynonymous mutations (identified in 5,248 viral genomes) are enriched in the S protein (**A**) and particular mutations *(e.g*., D614G) are frequent (occurring more than 20 times early in the pandemic) and retained in human, bat, and pangolin coronaviruses (**B**). None of the common variants are fixed in human populations. The tree of common variants is a maximum likelihood tree. The mutations are color coded by amino acid for taxa in the tree. Each colored tip in the tree is made up of one or more samples. The human samples are numerous, and the merging of many dots for many samples gives rise to the purple bar.

Shared ancestral polymorphisms in the gene trees also offer insights into natural selection^5^. Mutations in the coronavirus genome are nonuniform in their distribution along genes, such as the S protein encoding gene (Fig. 4A). This nonuniformity is displayed synchronously in both synonymous and non-synonymous mutations. For example, there is a great excess of nonsynonymous mutations between coronavirus Spike glycoprotein S1 domain and S2 domain. Mutations are particularly high in the bCov_S1_N domain. Some of the mutations are much more common in human coronaviruses.

Out of 90 nonsynonymous mutations (Fig. 4A), 5 of the variants were sequenced more than 20 times among human coronaviruses (Fig. 4B). One interesting feature of these common variants in the S protein shown is that they are retained polymorphisms shared between human, bat, and pangolin coronaviruses. None of these 5 common variants are fixed in human coronaviruses (Fig. 4B), although the probability of fixation in human coronaviruses is high^26^. Some of these retained S protein polymorphisms (e.g., P1263L) extend clear back to the MERS SARS outgroup. Another mutation (D614G) is preserved in all SARS-CoV-2 lineages, alpha, beta, gamma, and delta coronaviruses^27^. If the sample frame is increased to the end of March to over 41,910 coronavirus sequences, then 15 out of 17 common variants are shared between human, bat, and pangolin coronaviruses (Fig. S26). If one examines the mutations defining these 17 mutations, there is at least one additional associated mutation shared with bat or pangolin coronavirus in each of 10 of these defining mutations in the S gene (Table S1). The times to common ancestry of human, bat, and pangolin preclude incomplete lineage sorting as the explanation (Table S1). There are two alternate explanations. One possibility is natural selection. The other possibility is that there is still ongoing gene flow between human, bat, and pangolin coronaviruses.

Among these variants (Table S1), only one variant is at substantial frequency (D614G) early in the pandemic. During March of 2020, there were 28,005 copies of the variant recorded out of 38,110 sequences examined. Thus, its frequency was about 0.73 worldwide in March of 2020. Assuming the allele is neutral, the age of this allele from its origin at a frequency p = 1/2N_e_ = 1/2 × 4327 from Table S3 to a current frequency of x = 28,005/38,110 $\approx 0.73$ can be estimated as 14,777 generations $\pm$ 9204 generations^28^. Using the estimated generation length of 0.69 days^29^ in Table S3, the age of this D614G allele is about 10,196 $\pm$ 6350 days. If this variant arose near the beginning of the pandemic on December 8, 2019, the variant could be no older than 199 days (z = (199–10,196)/6350 = − 1.58), which is not significantly different from the predicted age of a neutral variant. The z-statistic doesn’t change much in value if SARS-Cov-2 was seeded into the human host with up to 20 copies of the D614G variant.

A more powerful test can be carried out for the neutrality of the D614G variant by considering its estimated allele frequencies over time in January (${\widehat{p}}_{0})$, February (${\widehat{p}}_{1}$), and March, 2020 (${\widehat{p}}_{2}$): (${\widehat{p}}_{0}$) 25/512 = 0.0488; (${\widehat{p}}_{1}$) 1,024/1,322 = 0.2254; and (${\widehat{p}}_{2}$) 28,005/38,110 = 0.7348. Under the random drift hypothesis there should be nondirectional changes about the estimated frequency ${\widehat{p}}_{0}$ = 0.0488. The alternative hypothesis is positive selection on the D614G variant. If drift alone explains the allele frequency changes, then the variance in estimated allele frequency has two components, the sampling component, ${VAR}_{s}({\widehat{p}}_{t})$ and the drift component (${VAR}_{d}({\widehat{p}}_{t})$ ^30,31^. The sampling component is given by binomial sampling variance for a proportion and the drift component, by population genetics theory^26^:

$$VAR\left( {\widehat{{p_{t} }}} \right) = VAR_{s} \left( {\widehat{{p_{t} }}} \right) + VAR_{d} \left( {E\left( {p_{t} } \right)} \right) = \frac{1}{{N_{t} }}p_{0} \left( {1 - p_{0} } \right) + \left( {\frac{{N_{t} - 1}}{{N_{t} }}} \right)(1 - {\text{exp}}( - t/gN_{e} )),$$

where g is the generation length and t is the time (0, 30, or 60 days). Using a normal approximation to the allele frequency estimates and a generation length of g = 0.69 days, the 95% confidence intervals are given by:

$$[\hat{p}_{0} \pm 2sd\left( {\hat{p}_{0} } \right);\hat{p}_{1} \pm 2sd\left( {\hat{p}_{1} } \right); \;\;p_{2} \pm 2sd\left( {\hat{p}_{2} } \right) = \left[ {\left( {0.03,0.07} \right); \, \left( {0.18,0.27} \right); \, \left( {0.67, \, 0.80} \right)} \right]$$

The confidence intervals are nonoverlapping—the null hypothesis of drift is then rejected in favor of positive selection. The same confidence intervals are obtained if the generation length is increased to g = 7 days.

The other unusual feature of the mutational profile is that the profile of synonymous and nonsynonymous mutations along the S-gene track each other very closely (Fig. 4A). One possibility is that early in the pandemic there is little selection acting on the Spike protein in humans in the absence of SARS-CoV-2 therapies. A second possibility is that an RNA virus has a completely different mutational profile that is dependent on its stem-loop structure of its genome secondary structure^32^, which could be the determining factor in its mutational profile rather than its reading frame. For example, the common variant (D614G) is located in a single nucleotide bulge of a stem-loop^32^. This second hypothesis is also examined in Fig. 4B.

## Discussion

Use of coalescent theory has allowed the development of a classification system for SARS-CoV-2, inference about ongoing gene flow between viral species, a reconstruction of the history of the pandemic, and evidence for selection early in the pandemic. In this framework the unit of population size is the infected host, and the connections in the coalescent, the path of transmission of the coronavirus. While individual gene trees provide a basis to classify viral taxa, the challenge is to resolve the discordancy between individual gene genealogies as they percolate through an organismal phylogeny as well the bias in divergence times between species when a single gene tree is relied on. To resolve this problem, a consensus species tree was reconstructed with a phylogenetic method developed here to solve the classification problem from the 11 individual gene trees in SARS-CoV-2.

In this study we showed discordant evolutionary histories confounded efforts to understand the origins of the SARS-CoV-2 pandemic; moreover, individual gene trees tended to overestimate the divergence time between species of coronaviruses by their common ancestor predating the species splits. A multi-species coalescent tree approach provides a more reliable estimate of the divergence time between species. For example, individual gene trees (Figs. S16–S21) placed the divergence time between species at about 60 years (1960), while multi-species coalescent theory placed the divergence more recently at about 3 years. We used the multispecies coalescent tree approach to estimate the SARS-CoV-2 phylogeny and test hypotheses of Covid-19 origins and early evolution. The species tree suggests that SARS-CoV-2 likely originated with a single introduction into humans. While bat species are the likely reservoir, multiple interspecies transmission events were likely involved in the generation of SARS-CoV-2, possibly including transmission through pangolin hosts.

Striking differences exist between the estimated divergence times of viral species (3 y) and their mammalian hosts (60 mya). For example, ongoing gene flow would have the effect of reducing the genetic distance between coronavirus species. We specifically tested this hypothesis by showing that bat and pangolin coronaviruses in the same locality were more closely related than those elsewhere (Fig. 3a).

Once the consensus species tree was reconstructed to represent the phylogeny of coronavirus genomes, the variation between human, bat, and pangolin coronaviruses was assessed using MERS sequences as an outgroup. The ancestral sequence of the root of the human clade was inferred (See Materials and Methods) and only differed by 2 nucleotides from the Wuhan sequence (Fig. 2). A bat coronavirus sequence (RaTG13) was the closest to human SARS-CoV-2 sequences in the consensus species tree^2^. The variation in bat SARS-CoV-2 sequences dwarfed that in pangolin coronavirus and human SARS-CoV-2 (Fig. S15). The time to most recent common ancestor of human, bat, and pangolin coronaviruses varies from 1960 to 1975 as estimated separately from 6 gene sequences in these genomes, in line with previous estimates^1,33^, but is more recent based on the multispecies tree coalescent. The dating estimates of the pandemic were placed in the context of a sensitivity analysis (Fig. S23).

The consensus species tree strongly implies viral gene flow between bat and pangolin coronaviruses because of the deep divergence of 60 million years between the pangolin and bat hosts. This raises the question of how human, bat, and pangolin coronaviruses are maintaining their shared polymorphisms. There are at least two possible explanations. One possibility is limited gene flow through recurring zoonotic transfers of virus. That would imply pandemics are recurring between human, bat, and pangolin without the need of invoking natural selection to explain the shared polymorphisms (Fig. 3). A second possibility is that selection by the immune systems of human, bat, and pangolin occurs on the same variants to produce convergent evolution (Fig. 4). Tests of neutrality suggest that the mutational profile observed early in the evolution of SARS-CoV-2 is the result of positive selection. This is coincidental evidence of epistasis due to the shared polymorphisms^33^. In either case there is striking preservation of shared polymorphisms between human, bat, and pangolin coronaviruses (Fig. 4).

The consensus species tree allowed a classification of the phylogeography of SARS-CoV-2. Using the lense of human, bat, and pangolin beta coronaviruses, all three clades lack spatial separation in China (Fig. S12), but are phylogenetically diverged, thus falling in the unusual category II of phylogeography^5^. There are few examples of phylogenetic discontinuity without geographic separation^34^.

Intraspecific phylogeography provides a lense on the origin and source of the 2020 pandemic as well as a predictive framework for the spread of SARS-CoV-2 and its relatives both under migration and selection.

### Limitations

The classification system here refers to the coronavirus taxa in different hosts as species. Biological Species Concept (BSC) or the Phylogenetic Species Concept (PSC) could be referred to as a definition of species^35^. Both have their limitations. Here the notion of species is based on a BSC-PSC reconciliation^35^. Testing directly for reproductive isolating barriers (RIBs) as in the BSC would raise ethical challenges. In contrast, the BSC-PSC concept relies directly on the concordance of multiple markers across RIBs and could be used to evaluate the species status of betacoronaviruses in human, bat, and pangolin hosts as more isolates are taken from bat and pangolin. There are now the phylogenetic tools to classify betacoronaviruses based on their genomes into a species tree (Fig. 2) or a species network with a reticulate view of evolution (Fig. S24) as these bat and pangolin viral genomes are added.

Neighbor joining (NJ) is one of the most widely used tree reconstruction methods and was used here to reconstruct the consensus species tree for its ability to scale computationally (see Materials and Methods). Neighbor Joining has been argued to be consistent and closely related to parsimony^36^. Simulations have been done to validate its use^37^. One experiment here was done to validate its application. Building on the NJ consistency result, the initial sample of 5,248 viral genomes was expanded to 40,028 genomes with no change in the consensus species tree as specified in Fig. S12 from NJst, provided ORF 10 sequences were set aside. A limitation here is not knowing how accurate NJst is relative to the method of maximum likelihood derived from a multi-species coalescent model.”

## Materials and methods

### Sequenced MERS sequences

Libraries, enrichment, and sequencing: Following the manufacturer's instructions, the extracted viral RNA was broken up and prepared for first strand synthesis using the NEBNext® Ultra II RNA First Strand Synthesis Module (NEB #E7771). The NEBNext® Ultra II Non-Directional RNA Second Strand Synthesis Module (NEB #E6111) was used in accordance with its instructions to perform the second strand synthesis process using 20 ng of the first strand cDNA. 1.8X SPRI beads (Beckman #B23318) were used to purify the double stranded cDNA. We utilized 50–100 ng of cDNA to generate the sequencing libraries using the Nextera Flex for Enrichment 8 × 12 Kit (Illumina #200027213) in accordance with the manufacturer's instructions, depending on the final cDNA yield of each sample. Equivalent amounts of the pre-enriched libraries were pooled in 12-plexes, followed by a concentration step using AMPure XP beads in accordance with established protocols. From the pre-enriched 12-plex libraries, the Illumina's Respiratory Virus Oligo Panel 12-plex (RVOP, Illumina #20042472) was used to enrich for ~ 40 common respiratory viruses, including SARS-CoV-2 using its hybridization and enrichment protocol. The enriched libraries were amplified then purified as described in the Illumina Kit’s manual. The quality of the final enriched libraries was assessed by measuring the concentration on the Qubit and estimating the size distribution on the Fragment Analyzer. The libraries were sequenced on the Illumina’s NextSeq 500 platform using the High Output flow cell and the paired-end 75 nucleotide. The accession for the MERS sequences in Dryad is https://datadryad.org/stash/share/im-2eUY0eVadwZyDeNf67InjenL-CeZKlErUaWemIaw.

### Sequence data collection

Beta-coronavirus (Beta-CoVs) genomes deposited in the Global Initiative on Sharing All Influenza Data database (GISAID) initiative from December 1, 2019 to April 17, 2020 were accessed through the EpiCov database^38^. We downloaded the publicly available metadata and pre-processed sequences on April 17, 2020 totaling 8,428 virus genomes from human, bat and pangolin hosts. Following filtering sequences with missingness, containing frame shift, and incomplete genomes of < 29kB, 5212 virus genomes passed filter for analysis. The open reading frames for each gene region were extracted based on the 5′ start and 3′ stop of each gene.

Archived other closely related Beta-CoV genome sequences were accessed from NCBI GenBank “nucleotide” database. Nucleotide sequences for each gene were extracted based on the available gene annotation information. Additional 5 MERS-CoV sequences were collected from and assembled using IRMA ((iterative refinement meta-assembler)^39^with a customized built coronavirus module. The virus annotation data is detailed in Supplementary data Table S1, some sequences only contains one or some of the genes in SARS-CoV-2. These sequences were collected and analyzed together with the Beta-CoVs genomes accessed from GISAID. After removing duplicate records, our final analysis is based on 5,248 sequences including 5209 isolates from human, 19 isolates from pangolin, and 16 isolates from bat and 5 MERS-CoV isolates. The sequence datasets for each of the 11 genes in SARS-CoV-2 and the full-genome sequence dataset were constructed.

To examine the sensitivity of some conclusions of this paper, on August 26, 2021, we downloaded all sequences from the early pandemic up to April 1, 2020, raising the sample to 41,910 coronavirus genomes which contain 920 SARS CoV-2 genomes from Italy. Following filtering sequences with missingness, containing frame shift, and incomplete genomes of < 29kB, 40,063 isolates passed the filter for analysis.10 additional bat coronavirus sequences were added into the analysis. The open reading frames for each gene were extracted as before and aligned.

### Alignments

We utilized amino acids to facilitate alignment of Beta-CoV nucleotide sequences that are isolated from different hosts. Briefly, nucleotide sequences were translated into protein sequences using package “EMBOSS Transeq”^40^ and then aligned in MAFFT v.7^41^ with default parameters. The codon-based nucleotide sequence alignments were further built with these alignments of proteins and corresponding nucleotide sequences using the program “PAL2NAL”^42^.

### Pairwise distances

We estimated the pairwise distances in each gene using the dist.dna function in the R package—‘ape’^43^. The raw model was specified as the evolutionary model. A heat map (Fig. S15) showed the genetics divergences between human, bat, pangolin, and MERS coronaviruses. Then the arithmetical average distances between there four groups were calculated.

### Gene tree estimation

Maximum likelihood (ML) phylogenetic trees were built from the 11 gene alignments using RAxML v. 8.2.11^19^, with the GTRCAT model (Figs. 1 and S1–S11). To reduce computational complexity, identical sequences were removed from the gene alignments. A rapid bootstrap analysis with 100 replicates was performed for each gene in RAxML with the command line raxmlHPC-SSE3-b seed1-p seed2-N100-mGTRCAT-s inputfile-n outputfile-k in which the seeds were generated at random for each gene. Then, the bootstrap gene trees were summarized by the majority rule consensus method implemented in a python script SumTrees v.4.0.0^44^. Branch lengths in each consensus gene tree from SumTrees^44^ are the mean branch lengths across all 100 bootstrap trees. So, the branch lengths on Fig. 1 are the mean of the expected number of substitutions per site across all 100 gene trees in the bootstrap sample. Finally, the identical sequences were added to the consensus tree with the bootstrap support value 100 and were visualized in FigTree v.1.4.4^45^. The only exception for building bootstrap gene trees was reconstructing the gene trees of 40,073 genomes of ORF 1a-b by the method of ML using IQTREE^46^. When summarizing bootstrap gene trees for 40,073 genomes of ORF1a-b and for 40,078 genomes of S, extended majority rule consensus method was implemented in RAxML with command line raxmlHPC -mGTRCAT -J MRE -z inputfile -n outputfile -p seed. The wall time for this reconstruction was 2 weeks for 100 parallel jobs for bootstrapping the ML method. The RAxML gene trees are available in Dryad https://datadryad.org/stash/share/im-2eUY0eVadwZyDeNf67InjenL-CeZKlErUaWemIaw.

### Reconstruction of early time line of human, bat, and pangolin coronaviruses with BEAST

We focused on the divergence times of Beta coronaviruses among human, pangolin, and bats, pangolin and bat isolates together with subsampled 10 SARS-CoV-2 sequences that were isolated during the early pandemic (before 2020-1-31) were included in this analysis. Time-measured phylogenetic reconstruction was performed using a Bayesian approach implemented in BEAST v.1.10.4^47^ with each coding gene (Figs. S16–S21). We took the mean value of the isolated period as the tip date when exact dates were not available. We performed analyses using a fixed starting gene tree topologies that were built from RaxML as described above. In the absence of any reasonable prior knowledge on the tMRCA of the datasets, we specified a simpler constant size population prior, applied an uncorrelated relaxed lognormal clock^48^ and GTR substitution model^49^ with a separate gamma model to accommodate inter-site rate variation^50^, and ran at least three analyses for 30,000,000 generations sampling every 3000 generations. Runs were assessed using Tracer v1.7.0^51^ to examine convergence, and a maximum clade credibility tree with median heights was created from 7500 trees after discarding a burn-in of 2500 trees.

### Gene tree network

A gene tree network (Fig. S13) was built from the 11 gene trees using SplitsTree4 (version 4.16.1), with the Super Network algorithm^52^. Since the gene trees are different for different genes, we combined the gene trees together to obtain a network relationship between taxa using the Super Network. To visualize the network, we randomly selected 502 taxa from human coronaviruses including Europe(202), Americas(189), Asia(54), Oceania(55) and Africa(2). We also included the genes of all the pangolin and bat coronaviruses to reconstruct the network.

### Species tree estimation

There were 100 distance matrices for each of 11 gene trees by bootstrap resampling. With 1100 (= 11 genes × 100 distance matrices) distance matrices generated from estimated gene trees, a consensus species tree was built by the majority rule method using SumTrees v.4.0.0^44^. More specifically, in each gene tree, distance between any two tips was calculated by finding the shortest path from one node to the other; *i.e*., finding the most recent common ancestor of two given nodes. All identical sequences were considered as a polytomy to eliminate the magnification of distance. For each bootstrap, based on the average distance matrix from 11 genes, a bootstrap species tree was built using the coalescent method NJst^53^ in which the neighbor joining was implemented in PHYLIP v.3.697^54^. Finally, the consensus species tree was summarized with 100 sets of bootstrap species trees by SumTrees (Fig. 2).

In order to validate our method with 40,000 + taxa dataset, neighbor joining species trees were reconstructed with the NJ function in ape in R^55^ to improve the efficiency. Then, the consensus species tree was summarized with the extended majority rule consensus method in RAxML with command line raxmlHPC-mGTRCAT-J MRE-z inputfile-n outputfile-p seed. For such a large dataset, ORF10 was removed to eliminate the influence of a missing ORF 10 gene for all bats and MERS samples. Such missing data would influence the average distance when other distances are not at same magnitude with ORF 10. This problem is not severe for a small dataset because of small non-identical sequences in each gene tree.

A faster distance matrix computing algorithm was developed for 40,000 + taxa compared with NJst function in the phybase library in R^56^, which could deal with smaller trees with 100 + taxa. First, the order of appearance for all taxa in a tree string was recorded. Next, we sequentially numbered all parentheses and recorded indexes of parentheses immediately before and after each taxon. From a short string consisting only of parentheses, the distance between the two taxa was 1 plus the number of unpaired parentheses between two of them. For example, for a small tree “((A, B), ((C, (D, E)), F));” the short string for the middle segment from B to F is “)((())”, after continually removing two paired parentheses “(())”, the short string only had “)(” left, indicating the distance from B to F is 3. Here, the short string in the middle segment of the two taxa are extracted according to the indexes recorded, all paired parentheses are regarded as siblings. By removing them we could find the shortest path.

### Species tree inference using StarBeast

The Bayesian species tree inference (Fig. 3) was conducted on human coronavirus (10 strains randomly selected from ~ 40,000 human coronavirus), bat coronavirus (26 strains), pangolin coronavirus (19 strains), and MERS coronavirus (5 strains) using StarBeast^24^. Since each of the four species (human, bat, pangolin, MERS) must have at least one strain present in the gene alignments, the genes with missing species were removed and only 5 genes (ORF 1a-b, S, M, N and ORF 3a) were used for the StarBeast analysis. The five genes constitute 92.9% of the whole coronavirus genome. General time reversible (GTR) model with the gamma (GAMMA) model of rate heterogeneity across sites is the most general substitution model in phylogenetic inference. We used a special case called the GAMMA Hasegawa, Kishino, Yano (HKY) GAMMA model, which produced similar results to the GTR model. We selected HKY + GAMMA as the site model and a strict clock model for the StarBeast analysis. The chain ran for 10 million generations, sampling every 1000 generations. The 10% burnin samples were discarded, and the remaining samples were summarized by Tracer^51^. To test whether gene flow may occur between bat coronavirus and pangolin coronavirus, 26 bat coronavirus strains were divided into two groups—20 strains sampled from China and 6 strains sampled from other countries, while the pangolin coronavirus (19 strains) were divided into a guangdong group (13 strains) and a guangxi group (6 strains). The Bayesian species tree inference was conducted on the new dataset using the same parameter settings described above.

### Bayesian phylodynamic analysis

Our large-scale sequence data, which contains 5,212 SARS-CoV-2 genomes, would significantly reduce the efficiency of a Bayesian analysis. We designed a subsampling strategy, and a subset with 200 genome samples was generated. A previous study shows that the coalescence of all sampled genomes can shift forward when analyzing the earliest samples as these basal lineages are lost and cease to propagate. To estimate tMRCA more accurately, it is important to include these early samples. In practice, we picked 39 samples which are collected prior to Jan 15th, and then we randomly selected 161 samples which were collected after Jan 15th.

Molecular clock analysis was conducted using a Bayesian Markov chain Monte Carlo (MCMC) approach in BEAST v1.10.4^57^. Two molecular clock assumptions and three coalescent models (i.e., constant population, exponential growth, and GMRF skyride were applied to evaluate the underlying population and evolutionary dynamics of the SARS-Cov-2 outbreak (Table S2 and Fig. S23). To investigate the divergence evolution processes in different genes, we applied the Hierarchical Phylogenetic Models which allowed each gene to have an unlinked substitution rate and clock rate. To facilitate convergence, an upper bound of 0.01 and a lower bound of 0.00001 substitutions/site/year was placed on the clock rate. For each model, five independent chains of 300 million generations were run, sampling every 30 thousand, and the first 20% were discarded as an equilibration run. The convergence and mixing were assessed in Tracer^51^, and chains were combined in LogCombiner. We also launched a sensitivity analysis on model/prior selection. We compared the posterior distribution inference by each model to find the sensitivity of tMRCA to the tree prior assumed (Fig. S23). Thus, it was important to perform model selection for phylogenomic inference.

To select the best-fitting model, we estimated the marginal likelihood which enabled the calculation of the Bayes Factor. We selected the path sampling / stepping-stone sampling, and we set the number of path steps to 100. For each step, BEAST was run with one-million-long MCMC chain to estimate the powered posterior.

### Reconstruction of Ancestral sequences

The ancestral sequence for each node were constructed with consensus gene trees and corresponding nucleotide alignments using the program IQ-Tree with “-asr” option^46^. The ancestral sequence for human SARS-CoV-2 were also estimated with the same approach.

### Identification of S gene mutations

The genetic variant of SARS-CoV-2 in spike protein region during the early of pandemic were identified with a custom python script by comparing the reconstructed ancestral sequence. Mutation Diagram was generated with a “Lolipop” software ^58^ and the distribution of mutations was estimated using R. A total of 5 representative non-synonymous single nucleotide polymorphisms (SNPs) with a frequency higher than 20 were identified and were further used in the polymorphism analysis of CoVs that are isolated from different hosts 0.150 human sequences without identified SNPs and 50 sequences that contained identified SNPs were subsampled using phylogenetic diversity analyzer (PDA) ^59^ to construct the phylogenetic tree in Fig. 4. Phylogenetic visualization was built with the R package “ggtree”^60^.

### Significance statement

The question is to understand the evolutionary factors, selection and migration, shaping the organismal pedigrees of SARS-CoV-2 and its relatives. To address this question, the consensus species tree of SARS-CoV-2 related coronaviruses was reconstructed from their 11 gene trees. Shared polymorphisms between species can arise in one of three ways as: (1) retained ancestral polymorphisms between human, bat, and pangolin coronaviruses ; (2) polymorphisms subject to a selective sweep (of a virulence gene(s)); (3) polymorphisms maintained by repeated gene flow. From the concordance of 11 gene trees the three hypotheses are distinguished. The species tree provides a unified classification system of SARS-CoV-2 in humans as well as inferences about SARS-CoV-2 origin and evolutionary factors operating on SARS-CoV-2.

## Supplementary Information

Supplementary Information.

## Data Availability

All analyses are available at https://github.com/michaelSkaro/On_origin_of_the_pandemic. The datasets generated and/or analysed during the current study are available in the Dryad repository, https://datadryad.org/stash/share/im-2eUY0eVadwZyDeNf67InjenL-CeZKlErUaWemIaw. The 5 new MERS-CoV coronavirus sequences used as an outgroup are also directly deposited in GenBank with accession numbers, OP654178, OP654179, OP712625, OP712624, and OP712623. The accessible persistent links for these accessions are: https://www.ncbi.nlm.nih.gov/nuccore/OP654178https://www.ncbi.nlm.nih.gov/nuccore/OP654179, https://www.ncbi.nlm.nih.gov/nuccore/OP712625, https://www.ncbi.nlm.nih.gov/nuccore/OP712624, https://www.ncbi.nlm.nih.gov/nuccore/OP712623.
