# Supplementary material for: The species coalescent indicates possible bat and pangolin origins of the COVID-19 pandemic
Source: Sci Rep. 2023 Apr 5;13:5571. doi: 10.1038/s41598-023-32622-4 (PMC10074375; doi:10.1038/s41598-023-32622-4)
Supplement: Supplementary file 1 — Supplementary Information. [file 41598_2023_32622_MOESM1_ESM.docx]

**Supplementary Information for**

The species coalescent indicates possible bat and pangolin origins of the COVID-19 pandemic.

Jialin Yang^1^, Michael Skaro^2^, Jiani Chen^2,3^, Duna Zhan^1^, Leke Lyu^2,3^, Skylar Gay^2^, Ahmed Kandeil^4,5^, Mohamed A. Ali^4^, Ghazi Kayali^6^, Kateryna Stoianova^7^, Pensheng Ji^1^, Magdy Alabady^7,8^, Justin Bahl^2,3,9,10^, Liang Liu^1^, and Jonathan Arnold*^5^

Statistics Department, University of Georgia, Athens, GA^1^; Institute of Bioinformatics, University of Georgia, Athens, GA^2^, Center for the Ecology of Infectious Diseases, University of Georgia, Athens, GA, Genetics Department, University of Georgia, Athens, GA^5^

^4^National Research Centre, Cairo, Egypt

^5^St. Jude Children’s Research Hospital, Memphis, TN

^6^Human-Link DMCC, Dubai, UAE

^6^University of Texas School of Public Health, Houston, TX

^7^ Georgia Genomics and Bioinformatics Center, University of Georgia, Athens, GA

^8^ Plant Biology Department, University of Georgia, Athens, GA.

^9^ Department of Infectious Diseases, College of Veterinary Medicine, University of Georgia, Athens, GA.

^10^ Department of Epidemiology and Biostatistics, College of Public Health, University of Georgia, Athens, GA.

*corresponding author: Jonathan Arnold.

**Email:**  [arnold@uga.edu](mailto:arnold@uga.edu)

Contents for Supplement: Fig.s S1, S2, S3, S4, S5, S6, S7, S8, S9, S10, S11, S12, S13, S14, S15, S16, S17, S18, S19, S20, S21, S22, S23, S24, S25, S26, and Tables S1, S2, and S3.

**Fig. S1**. The consensus gene tree of the ORF 1a-b genes has the human coronavirus clade monophyletic and the pangolin and bat coronavirus clades, polyphyletic, from 5,248 viral genomes. The gene tree was constructed by the method of maximum likelihood^1^. The consensus tree was constructed from a bootstrap sample of 100 trees. Human, bat, pangolin, and MERS-CoV coronavirus sequences are color-coded as black, red, orange, and dark orange (root), respectively. The 5 MERS-CoV sequences root the tree as an outgroup. The triangles represent the amalgamation of human SARS-CoV-2 lineages. Bootstrap support values are on branches. Scale bar for branch lengths is also given.

**Fig. S2**. The consensus gene tree of the S gene has the human coronavirus clade monophyletic and the pangolin coronavirus clade, paraphyletic, from 5,248 viral genomes. The gene tree was constructed by the method of maximum likelihood^1^. The consensus tree was constructed from a bootstrap sample of 100 trees. Human, bat, pangolin, and MERS-CoV coronavirus sequences are color-coded as black, red, orange, and dark orange (root), respectively. The 5 MERS-CoV sequences root the tree as an outgroup. The triangles represent the amalgamation of human SARS-CoV-2 lineages. Bootstrap support values are on branches. Scale bar for branch lengths is also given.

**Fig. S3**. The consensus gene tree of the ORF 3a gene has the human coronavirus clade monophyletic and the pangolin coronavirus clade, paraphyletic, from 5,248 viral genomes. The gene tree was constructed by the method of maximum likelihood^1^. The consensus tree was constructed from a bootstrap sample of 100 trees. Human, bat, pangolin, and MERS-CoV coronavirus sequences are color-coded as black, red, orange, and dark orange (root), respectively. The 5 MERS-CoV sequences root the tree as an outgroup. The triangles represent the amalgamation of human SARS-CoV-2 lineages. Bootstrap support values are on branches. Scale bar for branch lengths is also given.

**Fig. S4**. In the consensus gene tree of the E gene the human coronavirus lineage is paraphyletic and the pangolin and bat coronavirus lineages, polyphyletic, from 5,248 viral genomes. The E gene in human coronaviruses is paraphyletic respect to pangoline gene lineages. The gene tree was constructed by the method of maximum likelihood^1^. The consensus tree was constructed from a bootstrap sample of 100 trees. Human, bat, pangolin, and MERS-CoV coronavirus sequences are color-coded as black, red, orange, and dark orange (root), respectively. The 5 MERS-CoV sequences root the tree as an outgroup. The triangles represent the amalgamation of human SARS-CoV-2 lineages. Bootstrap support values are on branches. Scale bar for branch lengths is also given.

**Fig. S5**. For the consensus gene tree of the M gene the bat and human coronavirus clades form a monophyletic group from 5,248 viral genomes. The gene tree was constructed by the method of maximum likelihood^1^. The consensus tree was constructed from a bootstrap sample of 100 trees. Human, bat, pangolin, and MERS-CoV coronavirus sequences are color-coded as black, red, orange, and dark orange (root), respectively. The 5 MERS-CoV sequences root the tree as an outgroup. The triangles represent the amalgamation of human SARS-CoV-2 lineages. Bootstrap support values are on branches. Scale bar for branch lengths is also given.

**Fig. S6**. For the consensus gene tree of the ORF 6a gene the human and bat coronaviruses are polyphyletic and the pangolin clade, monophyletic, from 5,248 viral genomes. The gene tree was constructed by the method of maximum likelihood^1^. The consensus tree was constructed from a bootstrap sample of 100 trees. Human, bat, pangolin, and MERS-CoV coronavirus sequences are color-coded as black, red, orange, and dark orange (root), respectively. The 5 MERS-CoV sequences root the tree as an outgroup. The triangles represent the amalgamation of human SARS-CoV-2 lineages. Bootstrap support values are on branches. Scale bar for branch lengths is also given.

**Fig. S7**. The consensus gene tree of the ORF 7a gene has the human coronavirus clade being monophyletic and the bat and pangolin coronavirus clades being polyphyletic from 5,248 viral genomes. The gene tree was constructed by the method of maximum likelihood^1^. The consensus tree was constructed from a bootstrap sample of 100 trees. Human, bat, pangolin, and MERS-CoV coronavirus sequences are color-coded as black, red, orange, and dark orange (root), respectively. The 5 MERS-CoV sequences root the tree as an outgroup. The triangles represent the amalgamation of human SARS-CoV-2 lineages. Bootstrap support values are on branches. Scale bar for branch lengths is also given.

**Fig. S8**. The consensus gene tree of the ORF 7b gene has the human coronavirus clade being monophyletic and the pangolin and bat coronavirus clades being polyphyletic from 5,248 viral genomes. The gene tree was constructed by the method of maximum likelihood^1^. The consensus tree was constructed from a bootstrap sample of 100 trees. Human, bat, pangolin, and MERS-CoV coronavirus sequences are color-coded as black, red, orange, and dark orange (root), respectively. The 5 MERS-CoV sequences root the tree as an outgroup. The triangles represent the amalgamation of human SARS-CoV-2 lineages. Bootstrap support values are on branches. Scale bar for branch lengths is also given.

**Fig. S9**. The consensus gene tree of the ORF 8 gene has the human coronavirus clade being monophyletic and the bat and pangolin coronavirus clades being polyphyletic from 5,248 viral genomes. The gene tree was constructed by the method of maximum likelihood^1^. The consensus tree was constructed from a bootstrap sample of 100 trees. Human, bat, pangolin, and MERS-CoV coronavirus sequences are color-coded as black, red, orange, and dark orange (root), respectively. The 5 MERS-CoV sequences root the tree as an outgroup. The triangles represent the amalgamation of human SARS-CoV-2 lineages. Bootstrap support values are on branches. Scale bar for branch lengths is also given.

**Fig. S10**. The consensus gene tree of the N gene has the human coronavirus clade as monophyletic and the bat and pangolin coronaviruses, polyphyletic, from 5,248 viral genomes. The gene tree was constructed by the method of maximum likelihood^1^. The consensus tree was constructed from a bootstrap sample of 100 trees. Human, bat, pangolin, and MERS-CoV coronavirus sequences are color-coded as black, red, orange, and dark orange (root), respectively. The 5 MERS-CoV sequences root the tree as an outgroup. The triangles represent the amalgamation of human SARS-CoV-2 lineages. Bootstrap support values are on branches. Scale bar for branch lengths is also given.

**Fig. S11**. The consensus gene tree of the ORF 10 gene has the human coronavirus clade monophyletic from 5,248 viral genomes. The gene tree was constructed by the method of maximum likelihood^1^. The consensus tree was constructed from a bootstrap sample of 100 trees. Human, bat, pangolin, and MERS-CoV coronavirus sequences are color-coded as black, red, orange, and dark orange (root), respectively. The 5 MERS-CoV sequences root the tree as an outgroup. The triangles represent the amalgamation of human SARS-CoV-2 lineages. Bootstrap support values are on branches. Scale bar for branch lengths is also given.


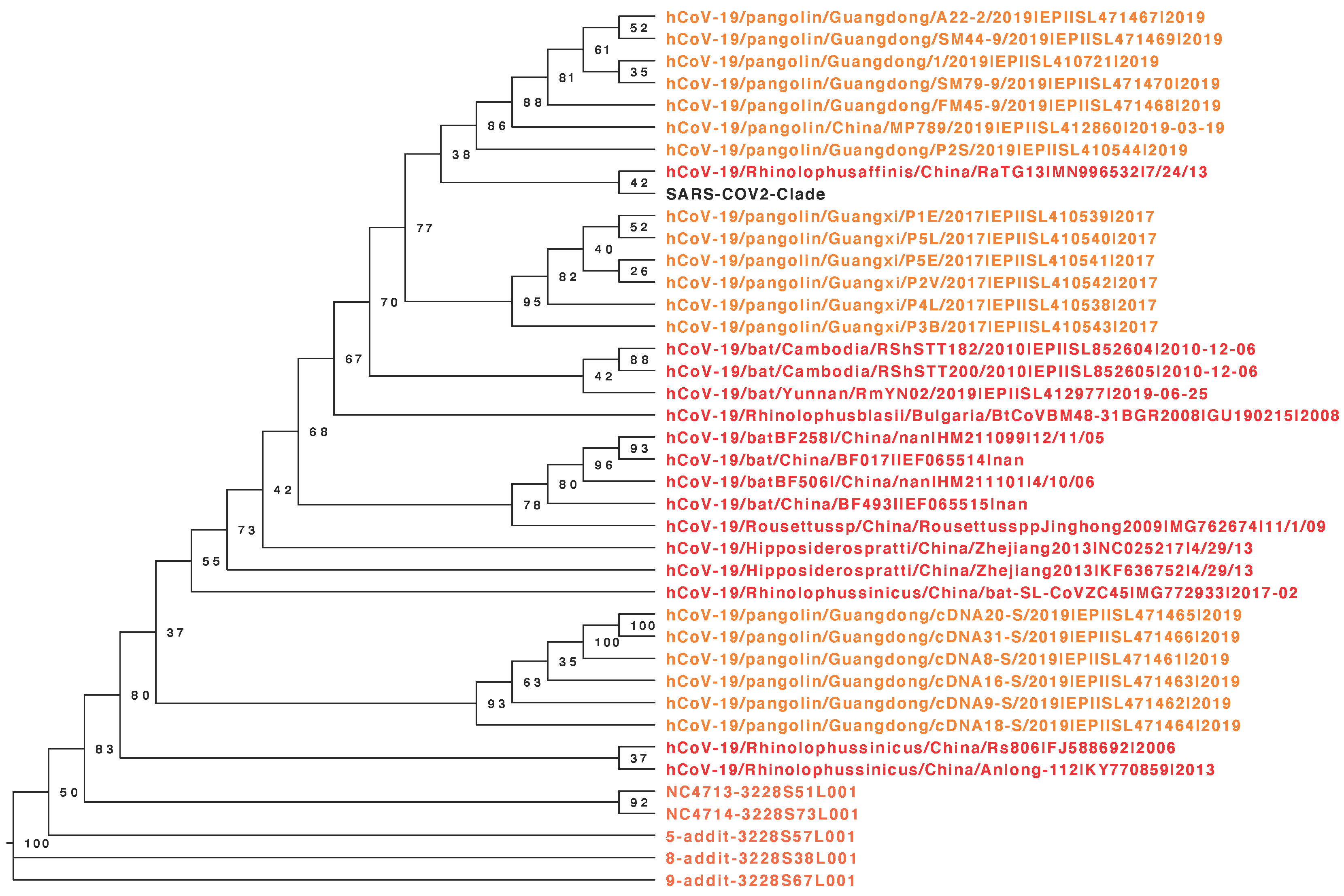


**Fig. S12**. The consensus species tree of the coronavirus genome has the human genome monophyletic and pangolin and bat coronavirus lineages polyphyletic from 5,248 viral genomes. The gene tree was constructed by the method of maximum likelihood^1^. The consensus tree was constructed from a bootstrap sample of 100 trees. Human, bat, and pangolin coronavirus sequences are color-coded as black, red, and orange respectively. The triangles represent the amalgamation of human SARS-CoV-2 lineages.

**Fig. S13.** C) A gene network reconstructed using the super network algorithm (Materials and Methods)^2^ cross-validates the placement of the bat coronavirus RATG13 as the closest to human coronavirus. Distances between 2 nodes in the network is the average number of mutations across genes. The closest human genome is labeled with Human in the network. The Human coronavirus sequences are labeled by regions in purple (Africa), yellow (Americas), red (Asia), green (Europe), and blue (Oceania). Bat coronavirus sequences are labeled in pink. Pangolin coronavirus sequences are labeled in orange. MERS coronavirus sequences are labeled in brown. In addition, the Bat coronavirus genomes, RATG and Yunnan (RMYN), both from Yunnan Province, are labeled as well as the closest Human sequence (Human) to them.


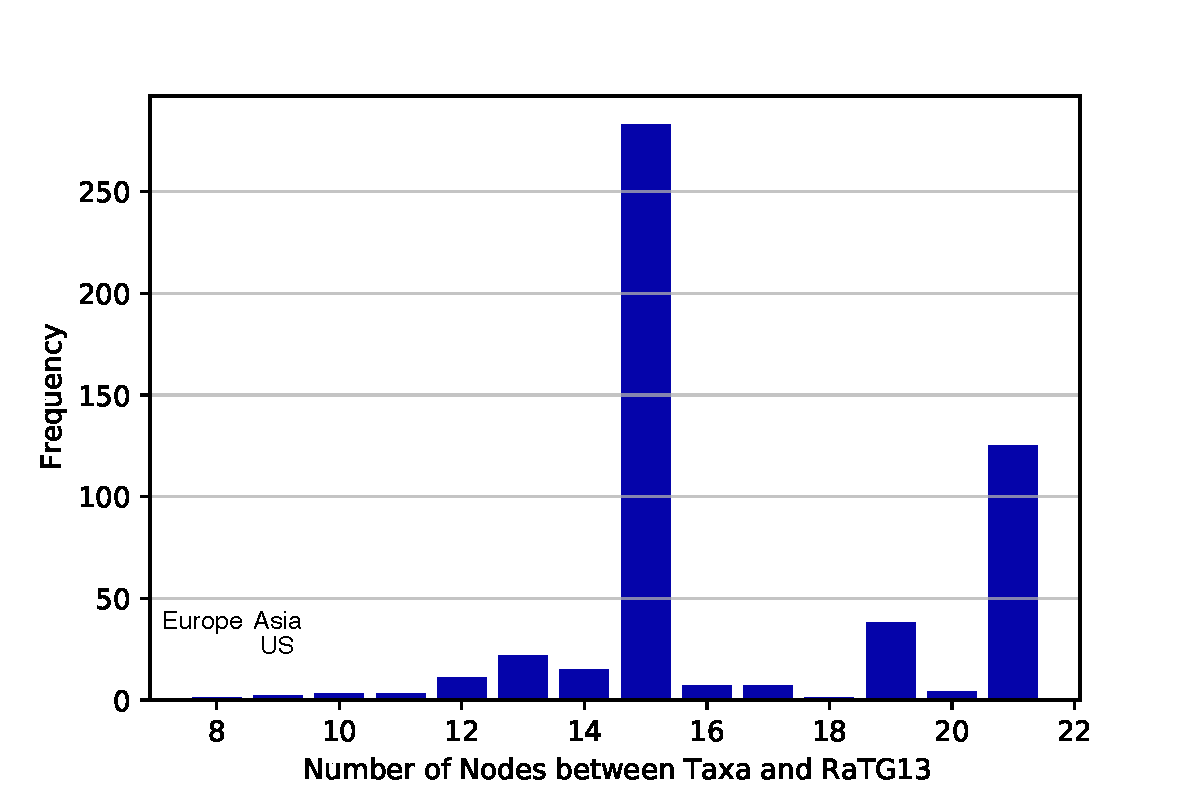


**Fig. S14**. The number of nodes in the super network (see Materials and Methods) that separates the RaTG13 bat lineage from a human coronavirus lineage in Europe, Asia, or the US is small (8-9 nodes) from 5,248 viral genomes. These human lineages are the closest to the bat coronavirus lineage (RaTG13).

**Fig. S15**. Bat lineages are highly divergent relative to the other taxa. A heat map is used to plot the nucleotide divergences between between human, bat, pangolin, and MERS coronaviruses among 5,248 viral genomes. The heat map was generated by ape^3^ (see Materials and Methods).

**Fig. S16**. Beast was used to reconstruct a time line using the earliest S gene sequences, such as Wuhan, and all bat and pangolin S gene sequences available. The earliest time for a common ancestor is 1949, but the 95% confidence band runs from 1900 to about 1986.

 **Fig. S17**. Beast was used to reconstruct a time line using the earliest ORF 1a-b gene sequences, such as Wuhan, and all bat and pangolin ORF 1a-b gene sequences available. The earliest time for a common ancestor is 1959, but the 95% confidence band runs from 1931 to 1981.

 **Fig. S18**. Beast was used to reconstruct a time line using the earliest E gene sequences, such as Wuhan, and all bat and pangolin E gene sequences available. The earliest time for a common ancestor is 1965, but the 95% confidence band runs from 1924 to 1995.

 **Fig. S19**. Beast was used to reconstruct a time line using the earliest M gene sequences, such as Wuhan, and all bat and pangolin M gene sequences available. The earliest time for a common ancestor is about 1954, but the 95% confidence band runs from 1897 to 1994.

 **Fig. S20**. Beast was used to reconstruct a time line using the earliest ORF 6 gene sequences, such as Wuhan, and all bat and pangolin ORF 6 gene sequences available. The earliest time for a common ancestor is 1962, but the 95% confidence band runs from 1906 to 2001.

 **Fig. S21**. Beast was used to reconstruct a time line using the earliest N gene sequences, such as Wuhan, and all bat and pangolin N gene sequences available. The earliest time for a common ancestor is 1970, but the 95% confidence band runs from 1933 to 1998.

**Fig. S22**. The molecular clock of SARS-Cov-2 varies with gene and region of the world based on 5,248 viral genomes. The genes are arranged in their order along the genome. Regions are color coded in the legend. The error bars represent 2 standard errors. These rates were computed based on a regression through the origin of SARS-CoV-2 divergences from the ancestral sequence on date of collection.

**Fig. S23**. Sensitivity analysis on estimate of time to most recent common ancestor in SARS-CoV-2 sequences under varied clock hypotheses. The posterior distributions were computed from BEAST (see Materials and Methods) under 6 varying hypotheses about the molecular clock. Some summary statistics for each hypothesis can be found in Table S2. An alternative date format is used to make it easy to read an increase in time. The date 2019-09-01 is Sep 1, 2019.

**Fig. S24**. The RATG13 coronavirus genome from BAT is still adjacent to the human coronavirus clade with an alternate network reconstruction method (RF-Net 2) for the data in Fig. 2^4^. The number of reticulations was preset to 2. The number of randomly sampled viral genomes is 715. Bats are colored in red, and pangolin, in orange.


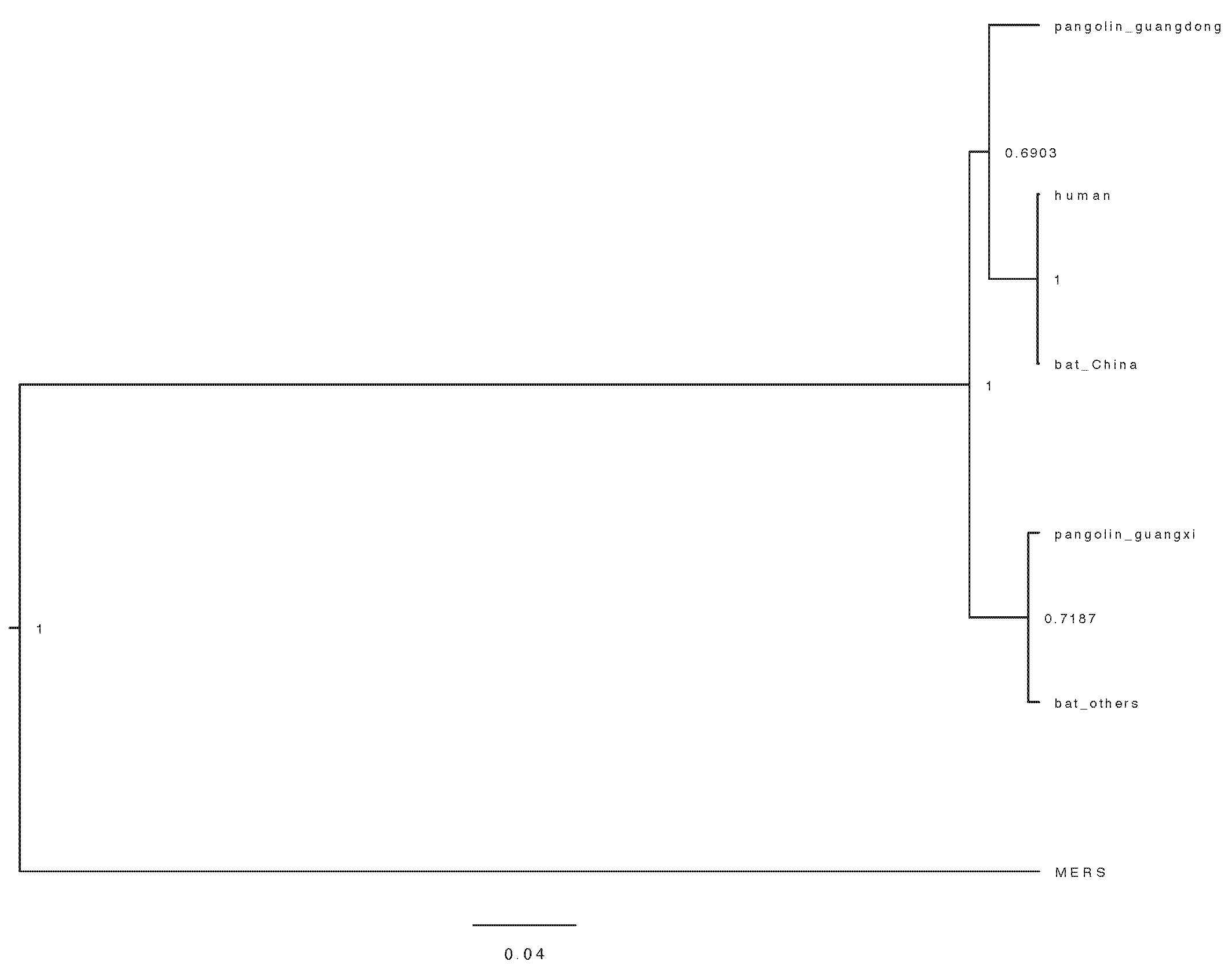


**Fig. S25**. Bat and pangolin coronaviruses from the same location are more closely related than those from elsewhere. The Bayesian species tree for human, bat_China, bat_others, pangolin_guangdong, pangolin_guangxi, and MERS coronavirus from StarBeast (see Materials and Methods). This is the same tree as **Fig. 3b** by StarBeast analysis, but posterior means have been added to this figure.

**Fig. S26**. 15 out of 17 mutations are shared among human, bat, and pangolin coronaviruses up to the end of March 2020 early in the pandemic (41, 910 coronavirus genomes sampled). None of these 18 common variants are fixed in human populations. The tree of common variants is a maximum likelihood tree. The mutations are color coded by amino acid for taxa in the tree. This figure is to be contrasted with Figure 6A only based on sequences available till April 17, 2020 (5,212 coronavirus genomes).

**Table S1**. The defining mutations of the variants below for gene (S) usually have an additional associated mutation shared with bat or pangolin coronavirus (BetCoV). D164G stands for a variant with amino acid D replaced by G in position 164.

| Type | "Human SARS-Cov-2" | "Bat BetaCoV" | "Pangolin BetaCoV" | "MERS" |
| --- | --- | --- | --- | --- |
| D614G | "D" "G" "N" | "D" "G" | "D" | "S" |
| P1263L | "P" "L" "S" | "P" | "P" | "P" |
| L5F | "L" "F" | "L" "S" "I" "A" "Q" | "F" "L" | "T" |
| D839Y | "D" "Y" | "D" "N" "E" "K" | "D" | "D" |
| A829T | "A" "T" | "A" "S" "T" | "A" | "A" |
| G1124V | "G" "V" | "G" "T" "S" | "G" | "A" |
| D936Y | "D" "Y" | "E" "S" "D" "Q" "K" "T" | "D" | "T" |
| L54F | "L" "F" | "Y" "F" "P" "L" "H" | "Y" "L" | "L" |
| Q675H | "Q" "H" "R" | "H" "T" "N" "I" "Q" "L" "V" | "Q" "X" | "D" |
| G1219C | "G" "C" "V" "S" | "G" "A" | "G" | "G" |
| T95I | "T" "I" "A" | "T" "I" "V" "Y" "F" | "T" | "P" |
| A222V | "A" "V" "S" "P" | "V" "A" "T" "P" "-" | "A" | "-" |
| L18F | "L" "F" "I" | "L" "I" "F" "-" "V" "Q" | "L" | "V" |
| N439K | "N" "K" | "A" "K" "R" "N" "I" "Q" | "N" "V" | "P" |
| S50L | "L" "S" | "L" "I" | "L" | "T" |
| R21I | "R" "I" "K" "T" | "R" "L" "K" "V" "G" "P" "T" | "R" | "Q" |
| H49Y | "H" "Y" | "V" "I" "H" "T" "A" | "V" "H" | "I" |

**Table S2**. Varied molecular clock hypotheses were evaluated in the coalescent using BEAST^5^ based on a sample of 200 viral genomes(see Materials and Methods), and the best hypothesis was the strict Gaussian Markov Random Field (GMRF) clock hypothesis based on the Bayesian posterior distribution. The log of the Bayesian posterior distribution is reported for 5 independent Markov Chain Monte Carlo runs. Both stepping stone and path sampling in MCMC were used with very similar results. Only the stepping stone sampling is reported here.

| Molecular clock hypothesis | Replicate 1 | Replicate 2 | Replicate 3 | Replicate 4 | Replicate 5 | Average |
| --- | --- | --- | --- | --- | --- | --- |
| Strict constant | -43956.71 | -43950.15 | -43961.09 | -43954.99 | -43951.75 | -43954.94 |
| Relaxed constant | -43966.73 | -43966.01 | -43958.54 | -43959.39 | -43961.06 | -43962.35 |
| Strict exponential | -43906.36 | -43904.80 | -43907.34 | -43903.62 | -43902.51 | -43904.92 |
| Relaxed exponential | -43916.61 | -43918.11 | -43912.20 | -43916.39 | -43912.69 | -43915.20 |
| Strict GMRF | -43900.64 | -43900.30 | -43900.99 | -43902.82 | -43903.78 | -43901.71 |
| Relaxed GMRF | -43915.31 | -43918.97 | -43920.09 | -43917.72 | -43916.76 | -43917.77 |

**Table S3.** The effective population size (N_e_) of human SARS CoV2 coronavirus can be estimated from the mean nucleotide divergence (p) for each gene (Figure 2), the rate (r) of the molecular clock, and the doubling time (d) of a coronavirus using 5,248 viral genomes. Absolute times to common ancestry can be further converted to times in generations using the measured doubling time of coronaviruses derived from the viral titer increase in cell lines of about 4 log_10_ counts/ml over 4 days^6^. This titer increase translates into a doubling time of .69 days. The nucleotide divergence p is computed by the 2-parameter Kimura Method^7^. As an example for the S protein, the calculation of the time in generations is t = (1/.00105) x (365/0.69) x 0.0032. The ration 365/0.69 was rounded to 528 days (per generation).

| gene | p | R (per site/lineage/yr) | d (in days)^6^ | t (in gen.s)^8^ | N_e_ |
| --- | --- | --- | --- | --- | --- |
| ORF1a-b | 0.0032 | 0.00117 | 0.69 | 1444 | 1444 |
| S | 0.0032 | 0.00105 | 0.69 | 1609 | 1609 |
| ORF 3a | 0.0049 | 0.00265 | 0.69 | 976 | 976 |
| E | 0.0039 | 0.00018 | 0.69 | 11440 | 11440 |
| M | 0.0057 | 0.00055 | 0.69 | 5472 | 5472 |
| ORF 6a | 0.00077 | 0.00043 | 0.69 | 945 | 945 |
| ORF 7a | 0.0051 | 0.00037 | 0.69 | 7278 | 7278 |
| ORF 7b | 0.0059 | 0.00019 | 0.69 | 16396 | 16396 |
| ORF 8 | 0.0029 | 0.00244 | 0.69 | 628 | 628 |
| N | 0.0040 | 0.00239 | 0.69 | 884 | 884 |
| ORF 10 | 0.00039 | 0.00039 | 0.69 | 528 | 528 |
| average |  | 0.00107 |  | 4327 +/-1609 | 4327 +/-1609 |

**References**

1 Stamatakis, A. RAxML version 8: a tool for phylogenetic analysis and post-analysis of large phylogenies. *Bioinformatics* **30**, 1312-1313 (2014). <https://doi.org:10.1093/bioinformatics/btu033>

2 Huson, D. H., Dezulian, T., Klopper, T. & Steel, M. A. Phylogenetic super-networks from partial trees. *IEEE/ACM Transactions on Computational Biology and Bioinformatics* **1**, 151-158 (2004). <https://doi.org:10.1109/TCBB.2004.44>

3 Paradis, E. & Schliep, K. ape 5.0: an environment for modern phylogenetics and evolutionary analyses in R. *Bioinformatics* **35**, 526-528 (2019). <https://doi.org:10.1093/bioinformatics/bty633>

4 Markin, A., Wagle, S., Anderson, T. K. & Eulenstein, O. RF-Net 2: fast inference of virus reassortment and hybridization networks. *Bioinformatics* **38**, 2144-2152 (2022). <https://doi.org:10.1093/bioinformatics/btac075>

5 Bouckaert, R. *et al.* BEAST 2: A Software Platform for Bayesian Evolutionary Analysis. *PLoS computational biology* **10**, e1003537 (2014). <https://doi.org:10.1371/journal.pcbi.1003537>

6 Kaye, M. SARS-associated coronavirus replication in cell lines. *Emerg Infect Dis* **12**, 128-133 (2006). <https://doi.org:10.3201/eid1201.050496>

7 Kimura, M. A simple method for estimating evolutionary rates of base substitutions through comparative studies of nucleotide sequences. *Journal of molecular evolution* **16**, 111-120 (1980). <https://doi.org:10.1007/BF01731581>

8 Avise, J. C., Ball, R. M. & Arnold, J. Current versus historical population sizes in vertebrate species with high gene flow: a comparison based on mitochondrial DNA lineages and inbreeding theory for neutral mutations. *Molecular biology and evolution* **5**, 331-344 (1988).
